# Fatal Diabetic Ketoacidosis and Suspected Non-occlusive Mesenteric Ischemia Induced by an Oral Glucose Tolerance Test: A Case Report

**DOI:** 10.7759/cureus.85391

**Published:** 2025-06-05

**Authors:** Takahiro Mamiya, Ryohei Isshiki, Naho Nojiri, Takahito Suzuki, Kento Kurita

**Affiliations:** 1 Education and Training Center, Handa City Hospital, Handa, JPN; 2 Endocrinology and Diabetes, Handa City Hospital, Handa, JPN

**Keywords:** critical care, diabetic ketoacidosis, diarrhea, fluid management, non-occlusive mesenteric ischemia, oral glucose tolerance test, septic shock, type 1 diabetes (t1d), early diagnosis

## Abstract

Advances in insulin therapy have contributed to improved outcomes in diabetic ketoacidosis (DKA). However, severe cases requiring vasopressor support continue to carry a poor prognosis. A 47-year-old man without a prior diagnosis of diabetes developed polydipsia following increased carbohydrate intake. Despite symptoms suggestive of hyperglycemia, he underwent an oral glucose tolerance test (OGTT) without a prior plasma glucose measurement. Immediately afterward, he developed shock with altered mental status. His plasma glucose was 1,740 mg/dL; pH, 7.091; and total ketone bodies, 13,320 μmol/L - confirming severe DKA. Despite aggressive management, he developed persistent diarrhea, followed by septic shock. Repeat computed tomography at 36 hours revealed extensive intestinal pneumatosis, which was not present on admission, suggestive of non-occlusive mesenteric ischemia (NOMI). He progressed to multiple organ failure and died 76 hours after admission. This case highlights the potential for an OGTT to precipitate severe DKA in patients with undiagnosed diabetes. Concurrent septic shock further complicates fluid management and increases the risk of NOMI. This case underscores three critical lessons: (1) plasma glucose should be measured before performing an OGTT in patients suspected of having diabetes; (2) vigilant fluid management and infection control are essential in cases of severe DKA with shock; and (3) persistent diarrhea, despite appropriate DKA treatment, may represent an early sign of bowel ischemia in patients with altered consciousness.

## Introduction

The mortality rate of diabetic ketoacidosis (DKA) has decreased from previously high levels to below 1% [1,2]. This reduction is attributed to advances in the understanding of DKA pathophysiology; widespread application of treatment guidelines emphasizing fluid resuscitation, insulin therapy, and electrolyte management; and advances in monitoring techniques [1-3]. However, in severe DKA requiring vasopressors, the mortality can reach 30.3% [2].

Oral glucose tolerance test (OGTT), a diagnostic procedure for diabetes involving glucose administration, is generally considered safe but can exacerbate hyperglycemia in undiagnosed diabetic patients. Previous case reports of DKA triggered by OGTT highlight the importance of measuring plasma glucose before glucose loading [4].

Non-occlusive mesenteric ischemia (NOMI), characterized by intestinal hypoperfusion without vascular occlusion, is a life-threatening complication that can occur as DKA progresses. In DKA, severe hyperglycemia causes hypovolemic shock that reduces mesenteric blood flow, while increased counterregulatory hormones promote mesenteric vasoconstriction, contributing to NOMI development [5].

Diagnosing NOMI is difficult because its clinical features, including abdominal pain, overlap with those of DKA. Furthermore, altered consciousness in DKA may prevent patients from reporting symptoms, making early recognition even more challenging. Given the high mortality associated with delayed diagnosis, early detection of NOMI is essential to improve outcomes [6,7]. Nevertheless, evidence regarding early clinical indicators of NOMI in DKA patients with impaired consciousness remains limited.

Here, we report a rare fatal case of undiagnosed type 1 diabetes, where OGTT triggered severe DKA, progressing to septic shock and suspected NOMI. This case uniquely highlights persistent diarrhea as a potential early warning sign for NOMI in vulnerable DKA patients - a clinical observation not previously emphasized.

## Case presentation

Medical history

A 47-year-old man had a family history of diabetes on his mother’s side, though the type was unknown. He smoked 10 cigarettes per day, occasionally consumed alcohol, and had no history of drug abuse. Previous health checkups had not revealed any evidence of diabetes.

Present history

Approximately three months before admission, he lost his job and subsequently increased his carbohydrate intake. During this period, he experienced persistent thirst. Three days before admission, he developed generalized fatigue. One day before admission, a local clinic detected 3+ glucosuria but did not measure his plasma glucose level. On the day of admission, the patient underwent an OGTT at the same clinic. Immediately afterward, he developed cold extremities and could barely stand or speak. The patient presented to our emergency department with altered consciousness.

Physical exam

On arrival, he was in shock, with a Glasgow Coma Scale score of 13. His vital signs were as follows: temperature, 35.0°C; blood pressure, 84/47 mmHg; heart rate, 92 beats/min; respiratory rate, 24 breaths/min; and oxygen saturation, 97% on room air. The patient was 165 cm tall and weighed 66.6 kg. His extremities were cold, and his oral mucosa was markedly dry. Abdominal examination revealed a soft abdomen without tenderness.

Six hours after admission, the patient developed repeated episodes of brown, watery stools. Although his impaired consciousness limited the reliable assessment of abdominal symptoms, physical examination revealed no new findings, and acute abdomen was not suspected at this time.

**Figure 1:**
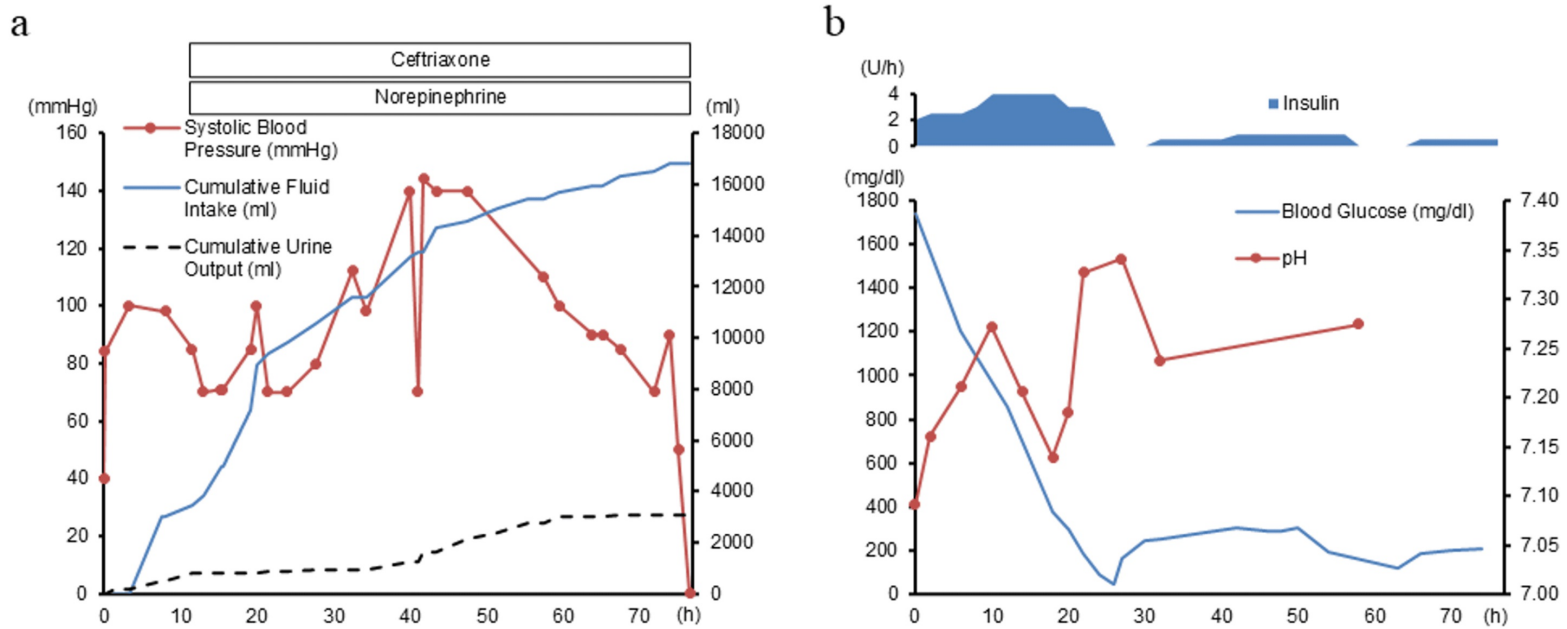
Clinical Course (a) Hemodynamic parameters and interventions: Systolic blood pressure (line with markers, left axis) remained unstable despite intravenous fluid resuscitation and norepinephrine administration. Cumulative fluid intake (solid line, right axis) and cumulative urine output (dashed line, right axis) show massive fluid administration, with poor urine response. Ceftriaxone was initiated 12 hours after admission for suspected infection. (b) Metabolic response: Continuous insulin infusion (upper area) rapidly reduced plasma glucose levels from 1,740 mg/dL (solid line, left axis). pH values (line with markers, right axis) are shown over time. The patient's condition progressively deteriorated, ultimately resulting in death 76 hours after admission.

Twelve hours after admission, his fever increased to 38.8°C. Despite receiving over 4,000 mL of fluids, he required norepinephrine to maintain blood pressure (Figure 1a). Thirty-six hours after admission, despite persistent altered consciousness, abdominal distension, and rebound tenderness became apparent.

Investigations

**Figure 2:**
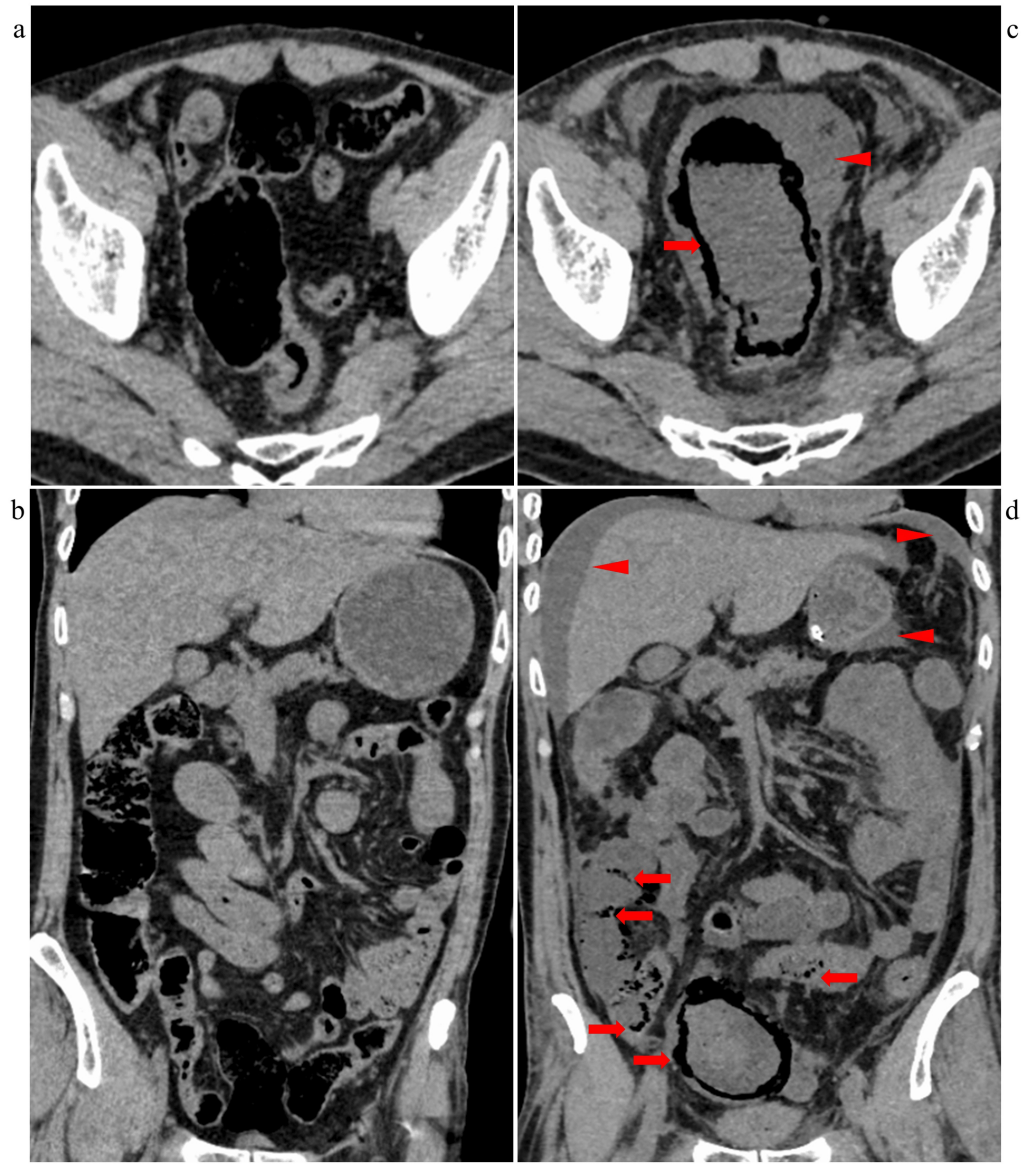
Computed Tomography Imaging of the Abdomen and Pelvis Computed tomography imaging of the abdomen and pelvis obtained on admission (a, b) and 36 hours after admission (c, d). Panels (a) and (c) show axial views, while panels (b) and (d) show coronal views. Extensive pneumatosis intestinalis (arrows) was observed from the rectum to the sigmoid colon, with additional involvement of the terminal ileum, suggesting non-occlusive mesenteric ischemia (c, d). Massive ascites (arrowheads) was also noted (c, d).

On arrival, laboratory tests revealed a plasma glucose level of 1,740 mg/dL, a pH of 7.091, and total ketone bodies of 13,320 μmol/L, confirming severe DKA [1,8]. His HbA1c was 10.6%, C-peptide level was 1.19 ng/mL, and anti-glutamic acid decarboxylase (GAD) antibody titer was 7.0 U/mL, suggesting type 1 diabetes (Table 1) [9]. His C-reactive protein (CRP) level was mildly elevated at 2.15 mg/dL. His renal function was severely impaired, with a blood urea nitrogen (BUN) of 89.7 mg/dL and creatinine of 4.88 mg/dL. His serum potassium level was 3.3 mEq/L, indicating mild hypokalemia. Although his amylase level was elevated at 623 U/L, neither physical examination nor abdominal computed tomography (CT) findings strongly suggested acute pancreatitis. Toxicology screening was not done. A non-contrast CT of the head, chest, abdomen, and pelvis was performed on admission to investigate other potential causes of shock and precipitating factors for DKA, revealing no significant abnormalities (Figures 2a-2b). These unremarkable CT findings contributed to a delay in the diagnosis of NOMI.

**Table 1:** Laboratory Data on Admission Abbreviations: PaCO₂, partial pressure of arterial carbon dioxide; HCO₃-, hydrogen carbonate; WBC, white blood cell; BUN, blood urea nitrogen; eGFR, estimated glomerular filtration rate; Na, sodium; K, potassium; LDH, lactate dehydrogenase; CK, creatine kinase; AST, aspartate aminotransferase; ALT, alanine aminotransferase; CRP, C-reactive protein; Anti-GAD antibody, anti-glutamic acid decarboxylase antibody

Test	Value	Reference Range	Unit
pH	7.091	7.350-7.450	-
PaCO₂	18.8	35.0-45.0	mmHg
HCO₃-	5.5	22.0-26.0	mmol/L
Anion gap	26.9	10.0-20.0	mmol/L
Lactate	2.2	0.50-1.90	mmol/L
WBC	9,200	4,000-8,500	/µL
BUN	89.7	8.0-22.0	mg/dL
Creatinine	4.88	0.60-1.10	mg/dL
eGFR	11	90-130	mL/min/1.73 m²
Uric acid	19.8	3.6-7.0	mg/dL
Na	124	138-146	mEq/L
K	3.3	3.6-4.9	mEq/L
LDH	178	38-110	U/L
CK	511	62-287	U/L
AST	17	13-33	U/L
ALT	16	6-30	U/L
Amylase	623	37-125	U/L
CRP	2.15	0.00-0.30	mg/dL
Plasma glucose	1740	70-109	mg/dL
HbA1c	10.6	4.6-6.2	%
C-peptide	1.19	0.61-2.09	ng/mL
Total ketone body	13,320	28.0-120.0	µmol/L
Anti-GAD antibody	7	0.0-5.0	U/mL

Twelve hours after admission, his CRP increased to 8.64 mg/dL, despite a partial improvement in his plasma glucose level and acidosis (Figure 1b). His mean Sequential Organ Failure Assessment (SOFA) score was 8. Despite receiving over 4,000 mL of fluids (Figure 1a), his lactate level increased to 4.5 mmol/L, indicating septic shock.

Thirty-six hours after admission, laboratory tests revealed an aspartate aminotransferase (AST) level of 380 U/L, alanine aminotransferase (ALT) level of 149 U/L, and creatine kinase (CK) level of 19,390 U/L, indicating a strong suspicion of acute abdominal pathology. Repeat non-contrast CT demonstrated extensive intestinal pneumatosis from the ileum to the rectum (Figures 2c-2d). Due to his unstable condition and worsening organ function, contrast-enhanced CT was not performed. Blood cultures obtained at 12 and 57 hours were negative.

Treatment

On arrival, we diagnosed severe DKA and initiated fluid resuscitation with 3,000 mL of normal saline over six hours, along with continuous insulin infusion at 2-4 units per hour (Figure 1) [1,8]. Intravenous administration of 80 mEq of potassium within two hours of admission increased the serum potassium level to 4.6 mEq/L, and it remained above 4.0 mEq/L until the patient's death.

Twelve hours after admission, suspecting a bacterial infection, intravenous ceftriaxone was initiated at 2 g/day (Figure 1a). Thirty-six hours after admission, after consulting the surgical and anesthesiology teams, surgery was deemed unfeasible due to the patient's hypotension and the extensive intestinal area requiring resection, making survival unlikely after surgery.

Outcome

The patient died 76 hours after admission. No autopsy was performed at the family’s request. NOMI was considered highly likely due to CT findings of pneumatosis intestinalis in both the superior and inferior mesenteric artery territories, despite the patient having few risk factors for thrombosis or embolism [10].

## Discussion

DKA risk after OGTT in patients with undiagnosed diabetes

The patient’s three-month history of excessive thirst and three days of fatigue suggested that diabetes might have already been present. Current clinical guidelines recommend initial diabetes screening using plasma glucose and HbA1c levels [11]. In patients with undiagnosed diabetes, performing an OGTT can exacerbate hyperosmolar dehydration and ketone production [8]. Previous case reports of DKA triggered by OGTT are extremely limited, with only isolated reports in pregnant women [4], underscoring the rare but clinically significant nature of our case. Hyperosmolar dehydration, caused by severe osmotic diuresis due to marked hyperglycemia, leads to intravascular volume depletion and reduced cardiac output. These hemodynamic changes can provoke mesenteric vasoconstriction and hypoperfusion, potentially contributing to the development of NOMI [5]. This risk is particularly pronounced in patients with undiagnosed type 1 diabetes. In our literature review of DKA-associated NOMI cases (Table 2) [5,12-20], type 1 diabetes accounted for 4 of 11 cases, with half of these cases (two of four) being newly diagnosed at presentation. This finding aligns with broader epidemiological data showing that DKA remains disturbingly common at the time of type 1 diabetes diagnosis [21]. In patients with undiagnosed type 1 diabetes, unrecognized insulin deficiency can cause the glucose load from the OGTT to readily result in severe hyperglycemia. Indeed, in this case, the OGTT appeared to accelerate the progression from undiagnosed diabetes to DKA and subsequently to NOMI. Given these risks, glucose screening before OGTT is essential in patients with suspected diabetes, especially type 1 diabetes.

**Table 2:** Reported Cases of Non-occlusive Mesenteric Ischemia With Diabetic Ketoacidosis in the Literature Abbreviations: CT, computed tomography; NOMI, non-occlusive mesenteric ischemia; T1DM, type 1 diabetes; T2DM, type 2 diabetes; LDH, lactate dehydrogenase; CK, creatine kinase; CRP, C-reactive protein

Reference	Year	Age	Sex	Diabetes Status	Chief Complaint	Initial Abdominal Findings	Diarrhea	Reason for CT and Other Imaging Suspecting NOMI	Outcome
[12]	2007	5	F	T1DM (newly diagnosed)	Lethargy, polyuria, polydipsia	Abdominal distention and mild tenderness	Not reported	Worsened abdominal distention and tenderness, persistent acidosis	Survived
[13]	2010	55	F	T1DM	Altered mental status	No abnormalities	Not reported	Onset of abdominal distention and tenderness, fever	Survived
[14]	2010	65	M	T2DM	Altered mental status	No abnormalities	Not reported	Onset of abdominal pain, worsened acidosis, elevated LDH and CK	Survived
[15]	2011	21	M	Newly diagnosed	Altered mental status, vomiting	Abdominal distention (no tenderness)	Not reported	Onset of abdominal pain, fever, elevated CK and CRP	Died
[16]	2013	47	M	Fulminant T1DM (newly diagnosed)	Altered mental status	Not reported	Not reported	Onset of abdominal pain, elevated CK	Survived
[17]	2013	59	M	T2DM (newly diagnosed)	Altered mental status, shock	No abnormalities	Not reported	Onset of abdominal pain, suspected septic shock	Survived
[18]	2013	65	M	T2DM (newly diagnosed)	Altered mental status, vomiting	No abnormalities	Not reported	Decreased consciousness and blood pressure, elevated CRP	Survived
[19]	2019	66	M	T2DM (newly diagnosed)	Dizziness, fall	Mild abdominal pain (no tenderness)	Not reported	Worsened abdominal pain	Survived
[5]	2022	13	F	T1DM	Altered mental status, vomiting, oliguria	No abnormalities	Slightly loose stools	Worsened abdominal pain, fever, elevated CRP	Survived
[20]	2022	60	M	Not reported	Altered mental status	No abnormalities	Non-bloody diarrhea for three days	Elevated lactate	Survived
[20]	2022	41	M	Not reported	Altered mental status	No abnormalities	Not reported	Onset of acute respiratory distress	Survived

Challenges in fluid management for severe DKA with septic shock

Managing fluid resuscitation in patients with both severe DKA and septic shock is complex. DKA typically results in a fluid deficit of 10% of total body weight [22]. In septic shock, increased vascular permeability and decreased peripheral vascular resistance further compromise hemodynamic stability. Despite administering approximately 16.8 L of fluid, the patient’s urine output was only 3.1 L, resulting in a large positive fluid balance (Figure 1a). However, the patient likely required more fluid than estimated due to persistent diarrhea, insensible losses from fever, and third-space fluid shifts. Since no pulmonary edema or heart failure was identified, effective circulating volume was probably still insufficient. Therefore, a more aggressive fluid resuscitation strategy might have been warranted.

Value of persistent diarrhea as an early warning sign

Diarrhea is an uncommon symptom of DKA [23]. When persistent diarrhea occurs despite improvements in blood glucose levels and acidosis resolution, clinicians should suspect serious abdominal complications, including NOMI. Patients with DKA with altered consciousness are particularly vulnerable to delayed diagnosis of abdominal complications due to their limited ability to report symptoms. In these cases, persistent watery diarrhea is one of the few directly observable clinical signs of potential complications [10]. Watery diarrhea persisted in this patient even before the development of clear peritoneal signs, potentially serving as an early indicator of NOMI. Literature reviews on DKA-associated NOMI cases have occasionally noted the occurrence of diarrhea (Table 2) [5,20]. However, such reviews have not emphasized their diagnostic significance or documented cases where diarrhea directly led to CT imaging (Table 2) [5,12-20]. In this case, although CT imaging was ultimately performed after peritoneal signs became apparent, earlier recognition of intestinal ischemia at the onset of diarrhea might have facilitated a timelier diagnosis and intervention. Since the mortality rate of NOMI is largely attributed to delayed diagnosis or misdiagnosis, earlier detection may improve patient survival [6,7].

Therefore, when unexplained diarrhea persists during DKA treatment, early imaging should be considered to exclude intestinal ischemia. Based on our experience, diarrhea should not be dismissed as an incidental symptom but rather recognized as a potential early warning sign of NOMI, even in patients with altered consciousness. This case uniquely demonstrates such recognition, and this awareness may help prevent diagnostic delays and improve clinical outcomes.

Limitations

A limitation of this study is the absence of autopsy confirmation for NOMI, potentially affecting the definitive diagnosis. Additionally, the generalizability is limited due to the single-case nature of the report.

## Conclusions

The patient likely had undiagnosed type 1 diabetes. The OGTT, performed without prior measurement of plasma glucose, precipitated rapid progression to severe DKA, followed by septic shock and suspected NOMI, ultimately leading to multiple organ failure and death. Clinicians should measure plasma glucose levels before conducting an OGTT in patients suspected of having diabetes, remain vigilant in fluid management for severe DKA with shock, and recognize persistent diarrhea despite ongoing DKA treatment as a potential early sign of bowel ischemia in patients with altered consciousness. Although the generalizability of this finding is limited by the single-case nature of the report, the observation that persistent diarrhea may precede overt signs of NOMI highlights a potentially valuable clinical clue. Further research is warranted to explore its utility in facilitating earlier detection of NOMI.
